# Serum Vitamin Levels, Cardiovascular Disease Risk Factors, and Their Association with Depression in Korean Women: A Cross-Sectional Study of a Nationally Representative Sample

**DOI:** 10.3390/medicina59122183

**Published:** 2023-12-15

**Authors:** Seon Mi Lee, Jong Chul Baek

**Affiliations:** 1 Department of Obstetrics and Gynecology, Korea University Anam Hospital, 73 Goryeodae-ro, Seongbuk-gu, Seoul 02841, Republic of Korea; tjsal4142@naver.com; 2 Department of Obstetrics and Gynecology, Gyeongsang National University Changwon Hospital, 11 Samjeongja-ro, Seongsan-gu, Changwon-si 51472, Republic of Korea; 3 Department of Obstetrics and Gynecology, Gyeongsang National University School of Medicine, Jinju 52727, Republic of Korea; 4 Institute of Medical Science, Gyeongsang National University, Jinju 52727, Republic of Korea

**Keywords:** depression, serum vitamin levels, cardiovascular disease risk factors, South Korean women, cross-sectional study

## Abstract

*Background and Objectives:* Serum vitamin levels, cardiovascular disease risk factors, and their association with depression is a complex issue that has been the subject of much research. Therefore, we investigated the relationship between vitamin A, B9, and E levels, cardiovascular risk factors, and depression in premenopausal and menopausal South Korean women. *Materials and Methods:* This cross-sectional study used the 2016–2018 Korea National Health and Nutrition Examination Survey data. Depression was assessed using a questionnaire to check for symptoms of depression or the Patient Health Questionnaire–9. Blood samples were collected from the antecubital vein in the morning after an overnight fast. Covariates were defined as self-reported physician diagnoses. Well-trained medical staff performed the standard procedures. Statistical analysis was performed using the complex sample analysis method of SPSS, using two separate logistic regression models (model 1: adjusted for age; model 2: adjusted for age, marital status, smoking, and alcohol consumption). *Results:* A total of 3313 women aged over 20 years were enrolled. The association between vitamin A levels and depression was as follows: lower levels of vitamin A were associated with an increased risk of depression in premenopausal women in model 1 and model 2. The levels of serum vitamins E and B9 were not correlated with depression in premenopausal and postmenopausal women. In the premenopausal group, depression increased in the obesity (model 1: *p* = 0.037; model 2: *p* = 0.047) and diabetes mellitus (model 1: *p* = 0.010; model 2: *p* = 0.009) groups. The menopausal group with depression had higher rates of stroke (model 1: *p* = 0.017; model 2: *p* = 0.039) and myocardial infarction (model 1: *p* = 0.004; model 2: *p* = 0.008) than the group without depression. *Conclusions:* Depression is correlated with lower blood levels of vitamin A in premenopausal women. Vitamin B9 and E levels were not associated with depression independent of menopausal status. Depression is associated with obesity and diabetes mellitus in premenopausal women and with stroke and myocardial infarction in postmenopausal women.

## 1. Introduction

Depression is now a common health condition. According to the World Health Organization’s 2020 fact sheet, more than 265 million people of all ages suffer from depression and approximately 800,000 people die each year from suicide due to depression, making it the second leading cause of death [1]. Depression is therefore not only a personal problem but is also associated with negative socio-economic outcomes. This trend is also seen in China and Japan in Asia, including South Korea, and the incidence of depression is particularly high in elderly women [2,3,4]. Recently, as interest in vitamins has increased, studies have been conducted to investigate the effects of different types of vitamins on the incidence of depression [5]. Vitamin A is involved in several biological processes including neurodevelopment and regulating neurotransmitters [5]. Vitamin E is an antioxidant that helps to protect cells from oxidative stress. The link between oxidative stress and depression has been explored [6]. Folate is involved in the synthesis of neurotransmitters such as serotonin, which plays a key role in the regulation of mood [5,7]. Because the serum levels of vitamins A, E, and B9 were presented in the Korea National Health and Nutrition Examination Survey (KNHANES) data from 2016 to 2018, we selected vitamins A, E, and B9 among the several types of vitamins. Some studies have suggested that high serum vitamin A levels not only increase depression but also increase suicide rates [5,8]. However, other studies have shown that depression is not correlated with vitamin A level [9]. Cross-sectional studies assess vitamin A levels and depression at a single point in time. They can provide valuable insights into the prevalence of depression and vitamin A deficiency in a population, as well as possible correlations. While some papers have found that vitamin E, a well-known potent antioxidant, is negatively correlated with depression [10,11], others have argued that there is no association between depression and vitamin E [6]. The evidence is inconclusive, and the relationship between vitamin E and depression is complex. Most studies have concluded that vitamin B9 is negatively associated with depression [5,8], but some papers have suggested that there is no association between vitamin B9 and depression [3]. The conclusions of the above papers are inconsistent, and as these studies were mostly conducted in Western populations, they may not be applicable to Asians. In Korea, vitamin intake and depression have been studied using KNHANES (Korea National Health and Nutrition Examination Survey) data [12], but serum vitamin concentrations and depression have not been studied. Therefore, we planned to investigate the association between the concentrations of vitamins A, E, and B9 and depression.

There are two types of risk factors for depression: fixed risk factors and modifiable risk factors. Age and gender are the included fixed factors [13]. According to the above-mentioned incidence of depression, elderly women are particularly susceptible to depression. This is due to the decrease in female hormones, which affects the activity of neurotransmitters such as serotonin, which causes the feeling of happiness [4]. We therefore divided the subjects into the premenopausal and menopausal groups to eliminate the confounding variables of menopause status that influence depression. The modifiable risk factors are smoking, alcoholic consumption, reduced physical activity, and medical conditions. According to recent publications, cardiovascular disease, which is included in the medical conditions of changeable factors, has a strong positive correlation with depression [1,14,15]. This is possible because cardiovascular disease and its risk factors, such as obesity, diabetes mellitus, hypertension, and dyslipidemia [16], and depression share pathophysiological characteristics, including inflammatory response and homocysteine metabolism [17]. The pathophysiology of atherosclerosis is multifactorial [18]. Therefore, the effect of exercise on the prevention of atherosclerosis is less clear. Plausible effects of exercise are modulated by dietary factors [18,19]. Therefore, we defined cardiovascular disease factors as a concept that includes stroke, myocardial infarction, obesity, hypertension, diabetes mellitus, and dyslipidemia. Similarly, some studies have found an association between high levels of cardiovascular disease risk factors, such as obesity and hypertension, and depression, while others have not. A bidirectional relationship between cardiovascular disease (CVD) and depression has been consistently demonstrated in many studies [14,15,17]. Risk factors such as a sedentary lifestyle, poor diet, smoking, and obesity are common to both depression and cardiovascular disease [19]. Oxidative stress and inflammation are associated with both cardiovascular disease and depression [10,20]. Antioxidant vitamins such as vitamins A and E can reduce oxidative stress, which may have an impact on depressive symptoms [20,21].

The KNHANES, which represents the South Korean general population, has the significance of being the first study investigating the association between the occurrence of depression, serum vitamin levels (vitamins A, E, and B9), and cardiovascular disease risk factors in adult Korean women.

## 2. Materials and Methods

### 2.1. Study Design and Setting

The KNHANES is a nationwide, population-based, cross-sectional survey designed to assess the health and nutritional status of the Korean population and to evaluate health policies and programs in Korea. The survey is conducted annually in 192 regions of Korea, with a sample size of approximately 10,000 people each year. The data are from the 7th Korea National Health and Nutrition Examination Survey (KNHANES). The KNHANES is conducted by the Korea Disease Control and Prevention Agency. As our study is based on data from the Patient Health Questionnaire (PHQ-9), which is administered every two years, we examined two years of data from the 2016 and 2018 surveys. Trained medical staff, dieticians, health interviewers, and medical technicians conducted every stage of the study either at a mobile screening center or in participants’ homes. Written informed consent from participants was approved by KNHANES. The study was conducted according to the guidelines of the Declaration of Helsinki and approved by the Institutional Review Board of Gyeongsang National University Changwon Hospital (protocol code: GNUCH 2021-09-007 and date of approval: 23 September 2021).

### 2.2. Participants

**Figure 1:**
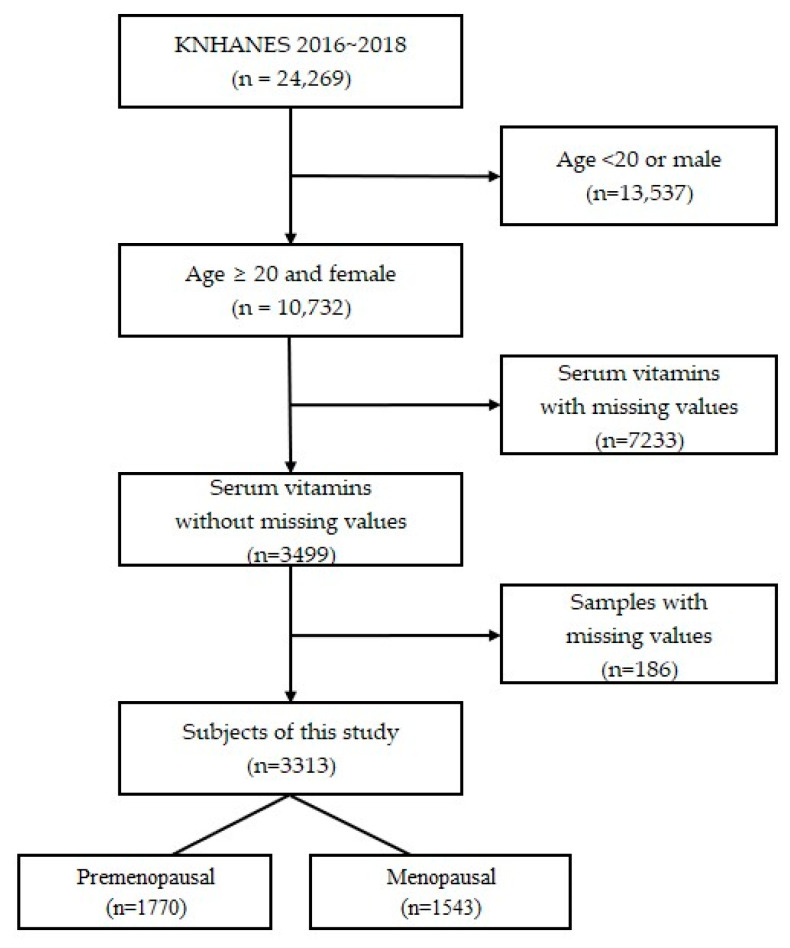
Flow diagram of the study population.

A total of 24,269 people who participated in the KNHANES between 2016 and 2018 were included in this cross-sectional study. According to the KNHANES, people older than 20 years were categorized as adults. In addition, the normal ranges of vitamins A, E, and B9 in the serum of adults were presented in the KNHANES by considering people who were 20 years of age or older as adults. Inclusion criteria were women over 20 years of age with serum vitamin A, E, and B9 testing, no missing values on the questionnaire, and serum vitamin levels. Exclusion criteria were samples younger than 20 years or male, serum vitamins with missing values, and missing samples. After application of our criteria, a total of 3313 women were selected, of whom those who had checked natural or artificial menopause in the questionnaire were classified as menopausal women and the rest were classified as premenopausal women. As a result, 1770 premenopausal women and 1543 menopausal women were invited to participate in the study (Figure 1).

### 2.3. Variables

#### 2.3.1. Definition of Depression

Depression, the dependent variable in this study, was defined as the presence of depressive symptoms; suicidal thoughts, suicide plans, or suicide attempts; a diagnosis of depression; or a score of ≥5 on the Patient Health Questionnaire–9 (PHQ-9; i.e., depressive symptoms). The PHQ-9 is a nine-item depression questionnaire that is a measure of the severity of depressive symptoms [16]. Each of the nine items is scored from 0 (not at all) to 3 (almost every day) according to how often the patient has experienced that symptom in the past two weeks. PHQ-9 scores ranged from 0 to 27, with scores interpreted as follows: 0–4, no depression; 5–9, mild depression; 10–19, moderate depression; 20–27, severe depression. In this study, ≥5 scores were judged as having depressive symptoms.

#### 2.3.2. Definition of Confounding Variables

The information contained in KNHANES was obtained through a self-administered health questionnaire. As the questionnaire, which was administered by well-trained survey staff members, was designed to obtain information on the physical health of the specimens and diagnosed diseases, we referred to the information, including stroke, myocardial infarction, hypertension, diabetes mellitus, and dyslipidemia, previously diagnosed by a physician as well as participant characteristics such as body mass index (BMI), marital status, smoking, alcohol consumption, and physical activity. Definitions of the confounding variables were as follows. BMI was defined as the person’s weight in kilograms divided by height in meters squared. The presence of a marital status was defined as married with a marital status, and the absence of a marital status was defined as single, divorced, separated, or widowed. Participants were defined as smokers if they reported either direct or passive smoking. Drinking was defined as an alcohol consumption of more than five complete drinks regardless of the type, soju or Western liquor, at a drinking party according to the KNHANES binge alcohol consumption classification. Physical activity was defined as participating in 150 min of moderate-intensity physical activity or 75 min or more of vigorous-intensity physical activity per week or equivalent physical activity according to the KNHANES aerobic physical activity practice item.

#### 2.3.3. Blood Sample Processing

The methods for measuring serum vitamin levels and other blood tests were as follows. To obtain the blood values given above, blood samples were taken from the antecubital vein in the morning after an overnight fast. Cholesterol, HDL, and LDL levels were measured enzymatically in all participants. Serum vitamin A and E levels were measured using a Chrom systems reagent high-performance liquid chromatography–flame ionization detection test method using Agilent 1200 equipment (Agilent Technologies Inc., Santa Clara, CA, USA). Serum vitamin B9 levels were measured by the chemiluminescent microparticle immunoassay test method using the ARCHITECT folate-only reagent and ARCHTECT i4000Sr equipment (Abbott, Abbott Park, IL, USA).

#### 2.3.4. Data Collection of Serum Vitamin Levels and Cardiovascular Disease Factors

Several factors potentially associated with depressive symptoms were selected as covariates in the present study based on clinical background and previous studies. Age and marital status were included as sociodemographic variables. Clinical variables included menopausal status, alcohol consumption, smoking, physical activity, and body mass index (BMI). The risk factors for cardiovascular disease were hypertension, diabetes mellitus, obesity, stroke, myocardial infarction, and dyslipidemia. We used the serum vitamin levels presented in KNHANES and collected from adults ≥20 years of age, with the following reference ranges: vitamin A, 0.30–0.70 mg/L; vitamin E, 5.00–20.00 mg/L; and vitamin B9, 3.1–20.5 ng/mL. Each of the cardiovascular disease factors was defined as follows. Stroke and myocardial infarction were defined as a previous diagnosis of stroke or myocardial infarction by a physician. Obesity was defined as BMI ≥ 25; hypertension as a previous physician diagnosis, systolic blood pressure ≥ 140 mmHg, or diastolic blood pressure ≥ 90 mmHg; diabetes mellitus as fasting glucose ≥ 126 mg/dL or HbA1c ≥ 6.5%; and dyslipidemia as high-density lipoprotein (HDL) cholesterol level ≤ 40 mg/dL or low-density lipoprotein (LDL) cholesterol level ≥ 130 mg/dL. Table 1 shows the general characteristics of the subjects in this study with respect to these factors.

### 2.4. Statistical Analysis

The participants in the study were stratified into two groups according to their menopausal status. We used a complex sample analysis because this study used KNHANES data extracted by a stratified colony sampling design rather than simple random sampling. Categorical variables were expressed as frequencies (*n*) with percentages (%), and continuous variables were expressed as means (M) with standard deviations. For the chi-square tests and the logistic regression analysis, the vitamin levels in the serum were divided into quartiles. The lowest quartile (Q1) of vitamins was used as the reference for the second quartile concentration (Q2), the third quartile concentration (Q3), and the highest quartile concentration (Q4). We performed multiple logistic regression analyses to determine the independent predictors of depression, with model 1 adjusted for age and model 2 adjusted for age, marital status, smoking, and alcohol consumption. For the above statistical analysis, IBM SPSS 25 was used, and statistical significance was determined based on a significance level of *p* < 0.05.

## 3. Results

### 3.1. Demographic Characteristics of Participants

The mean age of the total study population was 46.45 ± 0.30 years: 36.16 ± 0.24 years for premenopausal women and 62.01 ± 0.27 years for menopausal women. The incidence of depression in menopausal women was higher than that in premenopausal women, and the incidence rate was as follows. The incidence of depression in all subjects was 25.30% ± 0.90%: 24.90% ± 1.2% in premenopausal subjects and 26.10% ± 1.20% in menopausal subjects. Table 1 shows the overall characteristics according to depression in all subjects in this study. Overall, subjects with depression are more likely to be unmarried, smokers, drinkers, and have a history of stroke, myocardial infarction, diabetes mellitus, and dyslipidemia. They are also more likely to be obese. Premenopausal women with depression are more likely to be unmarried, smoke, and drink alcohol than women without depression. Menopausal women with depression are more likely to be unmarried, smokers, and have a history of stroke, MI, DM, and dyslipidemia.

### 3.2. Serum Vitamin Levels between Depressed and Non-Depressed Patients

Table 2 shows the mean and standard deviation of serum vitamin levels (vitamin A, vitamin E, and vitamin B9) in total subjects, premenopausal women, and menopausal women, stratified by depression status. Overall, there is no significant difference in serum vitamin levels between people with depression and people without depression, except for vitamin E in premenopausal women. Premenopausal women with depression have lower levels of serum vitamin E than premenopausal women without depression.

### 3.3. Association between Serum Vitamin Levels and Depression

Using the Complex Sample Crosstabs procedure, a cross-tabulation of depression frequency based on serum vitamin A, E, and B9 levels and divided into quartiles was created. As shown in Table 3, serum vitamin A levels were lower in premenopausal women with depression than in those without depression (χ^2^ = 11.507, *p* = 0.031). In premenopausal women, those with depression were more likely to have serum vitamin A levels in the lowest quartile (Q1) than in the second quartile (27.1% vs. 20.5%). In contrast, there was no significant difference in serum vitamin A levels between premenopausal women with and without depression in the other three quartiles (Q2, Q3, and Q4). In menopausal women, there was no significant difference in the serum levels of vitamins A, E, and B9 between women with depression and women without depression in any of the four quartiles.

We performed a multilevel logistic regression analysis by classifying model 1 adjusted for age and model 2 adjusted for age, marital status, smoking, and alcohol consumption to compare the influence of serum vitamin levels (vitamins A, E, and B9) and cardiovascular disease factors on depression. In univariate analysis, there was a significant association between vitamin A levels and depression, with lower levels of vitamin A being associated with an increased risk of depression in premenopausal women. In multivariable analysis, the association between vitamin A levels and depression remained significant correlation in both models (model 1: odds ratio (OR) 0.723, confidence interval (CI) 0.530–0.985, *p* = 0.04; model 2: OR 0.691, CI 0.505–0.946, *p* = 0.021). Serum vitamin E and B9 levels were not significantly correlated with the occurrence of depression in the premenopausal group (Table 4). In the postmenopausal group, serum vitamin levels (A, E, and B9) were not significantly correlated with the occurrence of depression.

### 3.4. Association between Cardiovascular Risk Factors and Depression

In the premenopausal group (Table 5), obesity increased the incidence of depression in both models (model 1: odds ratio (OR) 1.324, confidence interval (CI) 1.017–1.723, *p* = 0.037; model 2: OR 1.309, CI 1.003–1.708, *p* = 0.047). In addition, the risk of depression in diabetes mellitus was high in both models (model 1: OR 2.112, CI 1.193–3.739, *p* = 0.010; model 2: OR 2.123, CI 1.207–3.735, *p* = 0.009). In the menopausal group (Table 6), the incidence of depression in dyslipidemia was high after adjustment for age (model 1: OR 1.281, CI 1.000–1.641, *p* = 0.050), but no significant result was observed after correction for various covariates (model 2: OR 1.251, CI 0.976–1.603, *p* = 0.077). Menopausal women with depression had higher rates of stroke (model 1: OR 2.467, CI 1.178–5.167, *p* = 0.017; model 2: OR 2.214, CI 1.042–4.702, *p* = 0.039) and myocardial infarction (model 1: OR 7.301, CI 1.887–28.254, *p* = 0.004; model 2: OR 7.153, CI 1.686–30.342, *p* = 0.008) in both models.

## 4. Discussion

The results of this study show that serum vitamin E and B9 levels had no association with depression, but the association between vitamin A levels and depression was significant in premenopausal women. Obesity and diabetes mellitus were positively associated with depression in premenopausal women, and stroke and myocardial infarction were positively associated with depression in menopausal women. Studies of the association between serum vitamins and depression in Asian subjects are rare [6,7]. Previous studies in Western populations have generally reported that vitamin A levels are positively associated with depression [5,21,22,23] and that vitamin E and vitamin B9 levels are negatively association with depression [8,10,11,24,25,26,27,28,29]. Based on the conclusions of previous studies, the content is as follows. Vitamin A is essential for growth and development. It is necessary for the brain, especially the hippocampus, which is involved in emotions, learning, and memory [30]. Vitamin A is activated in a form called retinoic acid (RA). RA is a vital regulator in several neurobiological processes that are affected in depression [31]. There are a few possible explanations for the link between low serum vitamin A levels and depression. One possibility is that vitamin A plays a role in brain function and development. Vitamin A is essential to produce neurotransmitters, which are chemicals that allow brain cells to communicate with each other. Low vitamin A levels may disrupt neurotransmitter production and lead to depression symptoms [30]. Another possibility is that low serum vitamin A levels are a marker of inflammation. Inflammation is thought to play a role in the development of depression [31]. However, when vitamin A is supplied in excess, the beta-cis form of retinoic acid can be produced [32]. Because cis-retinoic acid has a much longer half-life (20 h) than the transform (0.9 h) and has a much higher affinity for the retinoic acid receptor, it decreases the neurotransmitters involved in emotions produced by the transform, thus increasing depression and the risk of suicide [32]. Research on the association between serum vitamin A concentrations and depression has yielded mixed results. While some studies have reported a positive correlation between low vitamin A levels and increased depression risk [5,8], others have not found a significant association [9]. Our results show that depression is correlated with lower blood levels of vitamin A in premenopausal women. Although the results do not support our findings, trans-beta-carotene is a precursor of vitamin A, which acts as an antioxidant in the brain and is mainly absorbed from vegetables and fruits. Therefore, fat-soluble vitamin A, which is mainly absorbed from animal foods, cannot be directly linked to depression [9].

Folate, also named vitamin B9, is involved in damaged DNA repair. Folate provides a methyl group for the synthesis of methionine. This is the immediate precursor of S-adenosylmethionine (SAM) [25]. SAM is the methyl donor in a myriad of methylation reactions in the brain and influences neurotransmitters (such as serotonin, dopamine, and norepinephrine) and hormone metabolism [8,26]. Several biological studies have linked depression to neurotransmitter and hormone disturbances [33]. A low level of folate contributes to hyperhomocysteinemia, which is correlated with the risk of depression [25,26,27,34]. This suggests that low folate levels might be associated with depression [8,11]. There were other studies which agreed with our own, showing that vitamin B9 failed to prevent depression. No association between depression and serum vitamin B9 levels was found in a study of 865 postpartum women by Miyake et al. [35] or in a study of a random sample of 6521 women aged >65 years by Penninx et al. [36]. A study of women in early pregnancy showed that folic acid intake did not protect against depression [37]. We speculate that the main reason for this discrepancy is that folic acid levels are a factor that varies greatly with dietary intake [36], and we did not take into account other factors affecting vitamin B9 levels, such as nutritional deficiencies or eating disorders, in our study group.

Vitamin E is a fat-soluble vitamin and antioxidant that plays a crucial role in protecting cells from damage caused by free radicals [38]. Free radicals are highly reactive molecules that can damage cell membranes, proteins, and DNA, contributing to the aging process and the development of various chronic diseases [10,38]. In addition to its antioxidant properties, vitamin E also has anti-inflammatory effects. Based on these theories, vitamin E may reduce depression [10,11].

Serum vitamin B9 and E were not associated with depression in this study. Owen found no association between serum vitamin E levels and depression, which is consistent with our findings [6]. Although depression appears to correlate positively with excessive brain free radicals, this is the result of age-related biological metabolic processes, not simply vitamin E deficiency. Furthermore, if depression is significantly more common because of vitamin E deficiency, then vitamin E supplementation should influence depression [1]. The results of this study are further support for the idea that there is no link between vitamin E and depression.

It is well known from many published papers that cardiovascular disease has a strong association with the incidence of depression [14,15,16,17,20], and similar results were obtained in this study. Previous studies have shown that although depression and cardiovascular disease are different conditions, they share pathophysiological features such as immune response, blood flow, glucose metabolism, and homocysteine metabolism [14,16,17]. Cardiovascular disease damages the vascular wall and increases vascular remodeling and atherosclerosis. The damage is exacerbated by risk factors such as hypertension, diabetes mellitus, dyslipidemia, smoking, and obesity [15,16]. In women, the consumption of fruit and vegetables was inversely associated with preclinical atherosclerosis [20]. High intakes of carotenoids and vitamins are associated with a diet rich in fruit and vegetables. Their antioxidant properties help prevent cholesterol and other lipids from oxidizing, reducing cardiovascular damage [39]. Reduced physical activity, in part through reduced nitric oxide bioavailability, impaired endogenous antioxidant defenses, increased the expression of ROS-generating enzymes, and has been shown to worsen cardiovascular function [40]. The excessive inflammatory and platelet responses promote thrombosis. As a result, stroke and myocardial infarction, which are the most common cardiovascular diseases, are caused [41]. In the initial phase of the inflammatory response, pro-inflammatory cytokines are activated. These include interleukin-1, interleukin-1b, and interleukin-6. Particularly, levels of interleukin-1 and interleukin-6 are also increased in depression [17]. Therefore, an increase in the inflammatory response that occurs in cardiovascular disease may contribute to the increase in depression. In addition, this oxidative stress causes an abnormality in fatty acid metabolism and increases homocysteine, one of the fatty acid metabolites [39]. When homocysteine accumulates above 11 μmol/L, the arginine moiety asymmetric dimethylarginine (ADMA) is produced, which acts as a competitive inhibitor of endothelial nitric oxide synthase (eNOS), inhibiting nitric oxide (NO) production and contributing to vasodilation [16]. In addition, high doses of homocysteine are converted to homocysteine-thioacetone or homocysteine H2O2 to increase endothelial toxicity, causing endothelial damage and vasoconstriction [16]. Homocysteine, which plays an important role in the exacerbation of cardiovascular disease, is also a factor involved in SAM metabolism, which is responsible for the synthesis of neurotransmitters such as dopamine, serotonin, and adrenaline [8]. Therefore, when homocysteine accumulates because of abnormal metabolism, the incidence of depression and cardiovascular disease increases.

## 5. Limitation of the Study

This large-scale study is the first to report the effects of serum vitamin levels on depression in a nationally representative sample of the Korean population, to the best of our knowledge. However, this study has several limitations. First, KNHANES was designed as a cross-sectional study, and longitudinal follow-up of all participants was limited. This did not allow causality to be established between depression and vitamin levels. Second, most of the serum levels of vitamins A, E, and B9 obtained in KNHANES were within the normal range, which limited our ability to investigate whether vitamin deficiencies or excesses were associated with depression. Third, dietary factors that affect serum vitamin levels, such as dietary habits, nutritional deficiencies, and eating disorders, were not assessed in our study. Fourth, we could not account for changes in serum vitamin concentrations that may occur due to the use of vitamin supplements, as there was no information on vitamin intake in the 2016–2018 KNHANES data used in this study. Fifth, we did not consider people’s history of taking drugs that affect folate metabolism, like methotrexate, anti-convulsants, and trimethoprim antibiotics. Sixth, serum vitamin concentrations are positively influenced by serum lipid levels. An accurate assessment of an individual’s vitamin E status requires an appropriate correction for this effect, but the effects on lipids were not analyzed in this study.

## 6. Conclusions and Recommendations

There is a complex and incomplete understanding of the relationship between serum vitamin levels and depression. Some evidence suggests that low serum vitamin A levels are linked with an increased risk of depression. Existing evidence suggests that low serum vitamin A levels may be a risk factor for depression, and our study found that Korean premenopausal women with depression were more likely to have low serum vitamin A levels than those without depression. Serum vitamin E and B9 levels are not significantly related with depression in our study. Among cardiovascular disease factors, obesity and diabetes mellitus are associated with depression in premenopausal women, and stroke and myocardial infarction are associated with depression in menopausal women. In other words, it is concluded that improving cardiovascular risk factors such as obesity or diabetes mellitus in premenopausal women and treating diseases such as stroke and myocardial infarction in menopausal women can help relieve depression. To confirm the results of the present study, further studies with data on more diverse serum vitamin levels and cardiovascular risk factors over a longer period of time in Korean women are needed.

## Figures and Tables

**Table 1:** Overall characteristics and depression in all subjects, premenopausal women, and menopausal women.

	Total (*n* = 3313)			Premenopausal Women (*n* = 1770)			Menopausal Women (*n* = 1543)		
	Depression			Depression			Depression		
	Yes (*n* = 815)	No (*n* = 2498)	*p*-Value	Yes (*n* = 418)	No (*n* = 1352)	*p*-Value	Yes (*n* = 397)	No (*n* = 1146)	*p*-Value
Age	46.4 ± 0.6	46.5 ± 0.3	0.898	34.6 ± 0.5	36.7 ± 0.3	**≤0.001**	63.4 ± 0.6	61.5 ± 0.5	**0.006**
Height	158.3 ± 0.3	158.5 ± 0.2	0.404	161.0 ± 0.3	160.6 ± 0.2	0.314	154.4 ± 0.4	155.3 ± 0.2	**0.034**
Weight	58.8 ± 0.4	58.3 ± 0.2	0.269	59.4 ± 0.6	58.5 ± 0.3	0.160	58.0 ± 0.6	58.1 ± 0.3	0.948
BMI	23.4 ± 0.1	23.2 ± 0.1	0.143	22.9 ± 0.2	22.7 ± 0.1	0.363	24.3 ± 0.2	24.1 ± 0.1	0.351
Marital status	486 (57.7)	1791 (69.5)	**≤0.001**	246 (55.8)	970 (67.5)	**≤0.001**	240 (60.5)	821 (72.6)	**≤0.001**
Smoking	212 (28.3)	413 (18.4)	**≤0.001**	141 (33.3)	266 (21.3)	**≤0.001**	71 (21.0)	147 (14.1)	**0.007**
Drinking	171 (24.9)	353 (17.0)	**≤0.001**	143 (35.6)	300 (24.2)	**≤0.001**	28 (9.6)	53 (6.0)	0.063
Physical activity	341 (44.4)	1077 (45.0)	0.816	209 (52.6)	647 (49.2)	0.253	132 (32.6)	430 (38.5)	0.072
Stroke	21 (2.7)	33 (1.0)	**0.002**	2 (0.4)	3 (0.2)	0.567	19 (5.9)	30 (2.2)	**0.003**
Myocardial infarction	12 (1.3)	4 (0.1)	**≤0.001**	1 (0.3)	0 (0.0)	0.083	11 (2.8)	4 (0.3)	**≤0.001**
Obesity	259 (30.9)	693 (26.7)	**0.025**	115 (24.9)	291 (21.3)	0.118	144 (39.4)	402 (34.9)	0.146
Hypertension	225 (23.2)	663 (22.7)	0.795	32 (6.9)	110 (7.9)	0.517	193 (46.7)	553 (45.5)	0.707
Diabetes mellitus	118 (13.1)	259 (9.4)	**0.006**	24 (4.5)	41 (2.7)	0.062	94 (25.6)	218 (19.8)	**0.043**
Dyslipidemia	272 (28.4)	662 (23.5)	**0.011**	68 (13.4)	158 (11.1)	0.222	204 (50.0)	504 (42.6)	**0.019**

BMI: body mass index. Values are presented as non-weighted *n* (weighted %) or mean ± standard error. The bold values are those with significant differences (*p* < 0.05).

**Table 2:** Serum vitamin levels according to depression in total subjects, premenopausal women, and menopausal women.

	Total (*n* = 3313)			Premenopausal Women (*n* = 1770)			Menopausal Women (*n* = 1543)		
	Depression (*n* = 815)	No Depression (*n* = 2498)	*p*-Value	Depression (*n* = 418)	No Depression (*n* = 1352)	*p*-Value	Depression (*n* = 397)	No Depression (*n* = 1146)	*p*-Value
Vitamin A	0.47 ± 0.007	0.46 ± 0.004	0.053	0.43 ± 0.008	0.42 ± 0.005	0.265	0.53 ± 0.010	0.51 ± 0.006	0.106
Vitamin E	13.7 ± 0.4	13.8 ± 0.2	0.874	12.2 ± 0.2	12.7 ± 0.1	**0.013**	15.9 ± 1.0	15.4 ± 0.2	0.639
Vitamin B9 (folate)	8.3 ± 0.2	8.2 ± 0.1	0.719	7.8 ± 0.2	7.7 ± 0.1	0.494	8.9 ± 0.2	9.0 ± 0.1	0.580

Values are presented as non-weighted *n* (weighted %) or mean ± standard error. The bold values are those with significant differences (*p* < 0.05).

**Table 3:** Serum vitamin levels in premenopausal and menopausal women with and without depression.

Variable		Premenopausal Women (*n* = 1770)				Menopausal Women (*n* = 1543)			
		Depression		χ^2^	*p*-Value	Depression		χ^2^	*p*-Value
		Yes (*n* = 418)	No (*n* = 1352)			Yes (*n* = 397)	No (*n* = 1146)		
Vitamin A	Q1	148 (27.1)	428 (72.9)	11.507	**0.031**	57 (26.1)	167 (73.9)	2.592	0.602
	Q2	111 (20.5)	419 (79.5)			81 (22.9)	274 (77.1)		
	Q3	78 (23.5)	283 (76.5)			106 (26.5)	315 (73.5)		
	Q4	81 (30.0)	222 (70.0)			153 (27.7)	390 (72.3)		
Vitamin E	Q1	144 (26.2)	429 (73.8)	2.373	0.583	75 (29.9)	180 (70.1)	5.358	0.301
	Q2	125 (25.7)	401 (74.3)			70 (21.7)	233 (78.3)		
	Q3	83 (23.8)	292 (76.2)			112 (26.1)	342 (73.9)		
	Q4	66 (22.0)	230 (78.0)			140 (26.9)	391 (73.1)		
Vitamin B9 (folate)	Q1	134 (25.2)	410 (74.8)	2.388	0.590	73 (27.3)	195 (72.7)	1.248	0.817
	Q2	115 (24.2)	369 (75.8)			91 (25.3)	284 (74.7)		
	Q3	89 (22.8)	319 (77.2)			109 (27.5)	309 (72.5)		
	Q4	80 (27.6)	254 (72.4)			124 (24.7)	358 (75.3)		

Vitamin A (mg/L): Q1: 0.08–0.35; Q2: 0.36–0.44; Q3: 0.45–0.54; Q4: 0.55–1.73. Vitamin E (mg/L): Q1: 1.65–10.51; Q2: 10.52–12.76; Q3: 12.77–15.81; Q4: 15.82–140.47. Vitamin B9 (ng/mL): Q1: 1.50–5.50; Q2: 5.60–7.70; Q3: 7.80–10.60; Q4: 10.70–40.00. Values are presented as non-weighted *n* (weighted %). The bold values are those with significant differences (*p* < 0.05).

**Table 4:** Logistic regression analysis of depression according to serum vitamins in premenopausal women using two-step multivariable logistic regression modeling.

	Premenopausal Women (*n* = 1770)									
	Univariate			Model 1 ^†^			Model 2 ^‡^			
		OR	(95%CI)	*p*	OR	(95% CI)	*p*	OR	(95% CI)	*p*
Vitamin A	Q1	1.00	Ref		1.000	Ref		1.000	Ref	
	Q2	0.693	(0.510–0.941)	**0.019**	0.723	(0.530–0.985)	**0.040**	0.691	(0.505–0.946)	**0.021**
	Q3	0.825	(0.580–1.173)	0.283	0.879	(0.613–1.261)	0.484	0.827	(0.571–1.198)	0.315
	Q4	1.151	(0.803–1.650)	0.443	1.307	(0.901–1.894)	0.158	1.140	(0.777–1.673)	0.503
	*p* for trend			0.603			0.263			0.677
Vitamin E	Q1	1.000	Ref		1.000	Ref		1.000	Ref	
	Q2	0.974	(0.705–1.344)	0.871	1.032	(0.746–1.429)	0.849	1.048	(0.751–1.462)	0.782
	Q3	0.878	(0.631–1.220)	0.438	0.969	(0.690–1.361)	0.855	0.992	(0.698–1.411)	0.966
	Q4	0.791	(0.561–1.115)	0.181	0.919	(0.638–1.323)	0.648	0.896	(0.617–1.302)	0.565
	*p* for trend			0.149			0.629			0.591
Vitamin B9 (folate)	Q1	1.000	Ref		1.000	Ref		1.000	Ref	
	Q2	0.947	(0.681–1.317)	0.746	1.016	(0.728–1.418)	0.924	1.069	(0.760–1.504)	0.700
	Q3	0.874	(0.627–1.217)	0.424	0.969	(0.685–1.370)	0.859	0.989	(0.694–1.411)	0.952
	Q4	1.128	(0.797–1.596)	0.497	1.251	(0.876–1.786)	0.218	1.380	(0.954–1.996)	0.087
	*p* for trend			0.715			0.319			0.164

Vitamin A (mg/L): Q1: 0.08–0.35; Q2: 0.36–0.44; Q3: 0.45–0.54; Q4: 0.55–1.73. Vitamin E (mg/L): Q1: 1.65–10.51; Q2: 10.52–12.76; Q3: 12.77–15.81; Q4: 15.82–140.47. Vitamin B9 (ng/mL): Q1: 1.50–5.50; Q2: 5.60–7.70; Q3: 7.80–10.60; Q4: 10.70–40.00. Y: yes; N: no; OR: odds ratio; CI: confidence interval; Ref: Ref. ^†^ Adjusted for age. ^‡^ Adjusted for age, marital status, smoking, and drinking. The bold values are those with significant differences (*p* < 0.05).

**Table 5:** Logistic regression analysis of depression according to cardiovascular disease risk factors in premenopausal women via two-step multivariable logistic regression modeling.

		Premenopausal Women (*n* = 1770)								
		Univariate			Model 1 ^†^		Model 2 ^‡^			
		OR	(95% CI)	*p*	OR	(95% CI)	*p*	OR	(95% CI)	*p*
Stroke	N	1.000	Ref		1.000	Ref		1.000	Ref	
	Y	1.732	(0.258–11.621)	0.571	2.303	(0.356–14.899)	0.381	2.119	(0.319–14.080)	0.437
MI	N	1.000	Ref		1.000	Ref		1.000	Ref	
	Y	∞			∞			∞		
Obesity	N	1.000	Ref		1.000	Ref		1.000	Ref	
	Y	1.228	(0.949–1.587)	0.118	1.324	(1.017–1.723)	**0.037**	1.309	(1.003–1.708)	**0.** **047**
Hypertension	N	1.000	Ref		1.000	Ref		1.000	Ref	
	Y	0.863	(0.552–1.349)	0.517	1.106	(0.691–1.770)	0.674	0.966	(0.593–1.567)	0.890
DM	N	1.000	Ref		1.000	Ref		1.000	Ref	
	Y	1.686	(0.970–2.930)	0.064	2.112	(1.193–3.739)	**0.010**	2.123	(1.207–3.735)	**0.** **009**
Dyslipidemia	N	1.000	Ref		1.000	Ref		1.000	Ref	
	Y	1.239	(0.878–1.750)	0.222	1.414	(0.986–2.028)	0.060	1.395	(0.980–1.986)	0.064

Y: yes; N: no; OR: odds ratio; CI: confidence interval; MI: myocardial infarction; DM: diabetes mellitus; Ref: Ref. ^†^ adjusted for age. ^‡^ Adjusted for age, marital status, smoking, and drinking. The bold values are those with significant differences (*p* < 0.05).

**Table 6:** Logistic regression analysis of depression according to cardiovascular disease risk factors in menopausal women via two-step multivariable logistic regression modeling.

		Menopausal Women (*n* = 1543)								
		Univariate		Model 1 ^†^			Model 2 ^‡^			
		OR	(95% CI)	*p*	OR	(95% CI)	*p*	OR	(95% CI)	*p*
Stroke	N	1.000	Ref		1.000	Ref		1.000	Ref	
	Y	2.765	(1.373–5.565)	0.004	2.467	(1.178–5.167)	**0.017**	2.214	(1.042–4.702)	**0.039**
MI	N	1.000	Ref		1.000	Ref		1.000	Ref	
	Y	8.198	(2.282–29.450)	0.001	7.301	(1.887–28.254)	**0.004**	7.153	(1.686–30.342)	**0.008**
Obesity	N	1.000	Ref		1.000	Ref		1.000	Ref	
	Y	1.216	(0.934–1.582)	0.146	1.178	(0.903–1.538)	0.227	1.162	(0.887–1.521)	0.275
Hypertension	N	1.000	Ref		1.000	Ref		1.000	Ref	
	Y	1.052	(0.806–1.373)	0.707	0.886	(0.666–1.180)	0.408	0.827	(0.620–1.105)	0.199
DM	N	1.000	Ref		1.000	Ref		1.000	Ref	
	Y	1.399	(1.010–1.938)	0.043	1.327	(0.957–1.840)	0.090	1.334	(0.954–1.864)	0.092
Dyslipidemia	N	1.000	Ref		1.000	Ref		1.000	Ref	
	Y	1.345	(1.051–1.722)	0.019	1.281	(1.000–1.641)	**0.050**	1.251	(0.976–1.603)	0.077

Y: yes; N: no; OR: odds ratio; CI: confidence interval; MI: myocardial infarction; DM: diabetes mellitus; Ref: Ref. ^†^ adjusted for age. ^‡^ Adjusted for age, marital status, smoking, and drinking. The bold values are those with significant differences (*p* < 0.05).

## Data Availability

The data evaluated in this study cannot be uploaded publicly due to legal restrictions, concerns for patient privacy, and third-party ownership of the data by the Korea National Health and Nutrition Examination Survey (KNHANES). However, the data are directly obtainable upon request, either by accessing https://knhanes.kdca.go.kr or by emailing knhanes@korea.kr.
